# Assessment of learning curves in complex surgical interventions: a consecutive case-series study

**DOI:** 10.1186/s13063-016-1383-4

**Published:** 2016-06-01

**Authors:** Olympia Papachristofi, David Jenkins, Linda D. Sharples

**Affiliations:** MRC Biostatistics Unit, Robinson Way, CB4 3EU, Cambridge, UK; Comprehensive Health Research Division, Leeds Institute of Clinical Trials Research, University of Leeds, 71-75 Clarendon Road, LS2 9PH, Leeds, UK; Departments of Surgery, Anaesthesia and Clinical Audit, Papworth Hospital, CB23 8RE,, Cambridge, UK

**Keywords:** Learning curve, Surgeon, Equipoise, Non-pharmacological interventions, Complex interventions

## Abstract

**Background:**

Surgical interventions are complex, which complicates their rigorous assessment through randomised clinical trials. An important component of complexity relates to surgeon experience and the rate at which the required level of skill is achieved, known as the learning curve. There is considerable evidence that operator performance for surgical innovations will change with increasing experience. Such learning effects complicate evaluations; the start of the trial might be delayed, resulting in loss of surgeon equipoise or, if an assessment is undertaken before performance has stabilised, the true impact of the intervention may be distorted.

**Methods:**

Formal estimation of learning parameters is necessary to characterise the learning curve, model its evolution and adjust for its presence during assessment. Current methods are either descriptive or model the learning curve through three main features: the initial skill level, the learning rate and the final skill level achieved. We introduce a fourth characterising feature, the duration of the learning period, which provides an estimate of the point at which learning has stabilised. We propose a two-phase model to estimate formally all four learning curve features.

**Results:**

We demonstrate that the two-phase model can be used to estimate the end of the learning period by incorporating a parameter for estimating the duration of learning. This is achieved by breaking down the model into a phase describing the learning period and one describing cases after the final skill level is reached, with the break point representing the length of learning. We illustrate the method using cardiac surgery data.

**Conclusions:**

This modelling extension is useful as it provides a measure of the potential cost of learning an intervention and enables statisticians to accommodate cases undertaken during the learning phase and assess the intervention after the optimal skill level is reached. The limitations of the method and implications for the optimal timing of a definitive randomised controlled trial are also discussed.

**Electronic supplementary material:**

The online version of this article (doi:10.1186/s13063-016-1383-4) contains supplementary material, which is available to authorized users.

## Background

Surgical interventions are complex multi-factorial procedures, which makes their formal statistical evaluation through randomised controlled trials (RCTs) more challenging compared to pharmaceutical interventions [1]. A pivotal aspect of surgical complexity concerns the abilities of the professionals delivering the intervention. A new procedure that is more technically demanding than the current standard of care may require a period of training to achieve satisfactory performance. Published guidance for the assessment of surgical interventions recognises the potential hazard of patient harm induced by surgeons at the initial phases of delivering a new intervention due to inexperience and emphasises the need for efficient training schemes to ensure satisfactory surgical standards [2].

Improvement in surgical performance over time is described as the learning curve and is expected to be most rapid in the initial stages of practice and then tail off over time. Three features were proposed by Cook et al. to characterise it: the initial level of performance, the learning rate measuring how quickly performance improves, and an asymptote or plateau representing the level at which performance stabilises [3].

Although learning curve modelling is in itself of interest, it is additionally useful as it may inform the design of RCTs. For instance, the requirement for a learning period influences the time of trial initiation. Early randomisation of patients may include cases undertaken during the learning phase, so that the imbalance in surgical expertise between the established and novel procedures may lead to biased estimates of treatment effects and ultimately to false conclusions against the novel procedure [3–5]. However, an RCT should begin only if equipoise still holds; if we wait too long, so that a pool of surgeons has reached their optimum skill level, it may be too late to randomise due to the surgeons forming strong opinions as to which treatment is best, despite the lack of scientific evaluation [6].

Formal statistical methods are required to estimate the optimal timing of an RCT assessment in the presence of learning. The timing of trial funding applications must also consider the duration of training for participating surgeons [7]. Furthermore, reliable estimation of the learning period and training required is also necessary for estimating the cost-effectiveness (monetary and clinical) of the new intervention [8].

Currently adopted approaches to learning curve accommodation include expert mentoring and completion of a predetermined number of training cases by each surgeon [3, 9]. However, performing a pre-specified number of operations does not guarantee the learning curve will be surpassed [3, 5]. Surgeons learn at different rates, their learning process may be influenced by external factors, and extended learning may be needed for complex procedures, which may not be finalised prior to trial initiation [5]. Notably in multicentre studies, different training and mentoring schemes between centres may result in different learning effects across both centres and surgeons [3, 10].

Further, as learning curves for many procedures are not formally quantified, existing criteria for trial participation may be based on poor evidence. Two systematic reviews of methods used to assess surgeon learning indicate that the majority of studies involving some statistical analysis employ exploratory or descriptive methods, which are often inefficient and are suitable only for identifying the existence of learning but not for estimating learning curve parameters [7, 11]. The use of formal, statistical modelling methods is infrequent and limited to estimating, at most, the three features described by Cook et al. [3]. Such models at least account for confounding factors but nevertheless cannot estimate the learning period.

No process for estimating the endpoint of the learning period was found in the literature, with recommendations based on either expert opinion or simple exploratory methods.

In this study we aim to develop a formal methodology to inform the timing of a definitive RCT in the presence of learning. A novel two-phase model estimating the time taken to reach the plateau in skill is developed. The methodology is illustrated using a high-risk cardiac surgery cohort undergoing pulmonary endarterectomy.

## Methods

### The two-phase model

Suppose we have a single case series for a novel intervention and we are interested in using formal statistical methods to determine the time point of learning completion. Our approach is to view the series of measurements in two phases:

1. the cases occurring during the learning phase, represented by the (non-linear) decreasing section of the curve and,
2. the cases occurring after the final performance level is reached, represented by the asymptotic part of the curve.

The point at which the split between these two phases occurs represents the number of cases at which the asymptote is reached and, naturally, the learning period duration. We refer to this as the two-phase model.

To model the learning phase, we propose curve-fitting through generalised (non-)linear models as they are simple to fit in standard statistical software, they allow for inclusion of confounding factors and they are flexible in that they include a wide range of functional forms and response variables, conditional on appropriate link functions. This approach is also an intuitively attractive method as we have a priori an idea of the expected shape of any learning curve [12]. Finally, the approach allows the exploration of individual surgeon differences.

We note that the split point can be estimated only for surgeons who have performed a sufficiently long series and have reached their plateau in the observed data. It is possible that some surgeons will reach a plateau that is subpar compared to the expertise levels required by the surgical community; stability in performance does not ensure competence [13]. The lack of guidelines indicating the clinically important distance from the asymptote has been highlighted previously [7]. We recommend eliciting expert opinion on the final surgeon competence and using it to interpret the two-phase model. In the next section, we discuss methods for establishing whether the desired skill level has been achieved.

### Assessment of competence

**Fig. 1:**
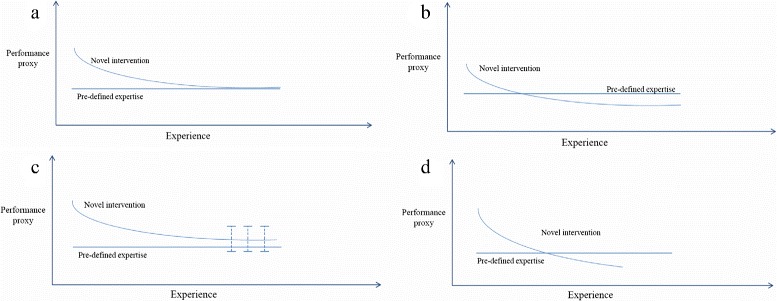
Learning curve scenarios for RCT randomisation (low values on the y-axis represent superior performance). Panels **a** and **b** depict scenarios where both a pre-defined expertise level and a learning plateau are reached; **c** depicts a scenario where the pre-defined expertise level is not achieved, and **d** a scenario where the learning plateau is not reached

Figure 1 presents four potential scenarios in which surgeons could be included in a Phase III RCT. Figure 1a depicts a surgeon who has performed enough procedures to have reached the asymptote and his plateau matches the predefined expertise level required; this is the value that should be, at the very least, achieved by a surgeon to be eligible for trial participation. Thus, the split point can be estimated and the plateau reached justifies sufficient expertise for the surgeon to be randomised in a definitive RCT.

In Fig. 1b, if the observed surgical series is sufficiently long to have reached the asymptote and the surgeon’s final skill level is superior to the preset expertise level, we can assume expertise has been achieved. However, the surgeons under study should be compared to the surgeons whom the required expertise estimate was based on, as it is likely that the performing surgeons are innovators and, thus, learn faster and accomplish superior final skill levels to those expected when the procedure was disseminated to the general community.

Figure 1c introduces the more complex scenario where the surgeon reaches a plateau in knowledge during the observed series, but the final skill level is inferior to the predefined skill level required. A pragmatic approach might be to accept that there will be heterogeneity in the final skill levels of surgeons and allow the trial to start as soon as the surgeon-specific skill level has stabilised. A more cautious approach might be to include in a trial only surgeons for whom the 95 % confidence intervals at the asymptote include the predefined expertise level. However, such an approach will be subject to type II error if the model parameters are estimated imprecisely. The final decision will depend on the nature of the measure of performance and the stage of the evaluation.

Finally, Fig. 1d depicts a case where the learning curve plateau is not reached but the learning curve has crossed the required skill level. This surgeon could still be considered as sufficiently experienced for trial participation but the treatment effect estimate may be underestimated by the RCT.

### Proxies for learning

One issue in this context is the choice of robust proxy outcomes for learning. Since surgeon skill cannot be directly quantified, patient outcomes (e.g. survival and post-operative complications) and surgical process outcomes (e.g. operative duration and blood loss) are used as surrogates [13]. These are analysed as a function of the number of operations completed, with improvements in measurements suggesting a gain in expertise. Patient outcomes should be preferred as they directly concern the success of the procedure in improving patient health. However, as these are typically dichotomous rare events, prohibitively large training series are often needed to model the learning curve [7, 10]. Process outcomes, for which more data are available, are widely used despite being poor representations of surgeon skill. For instance, a decrease in operative duration does not necessarily imply an improvement in performance as it may increase the risk of complications [7]. Additionally, factors such as case mix and team effects may influence the outcome and distort assessments [7]; longer operation times can be falsely interpreted as surgeon learning when they may be due to an inexperienced theatre team.

### Illustrative dataset

We demonstrate the methods using data from the UK national referral centre for pulmonary endarterectomy (PEA), a novel (at the time of data collection), highly technical surgical intervention for treating chronic thromboembolic pulmonary hypertension [14], for which no other surgical treatment is available [15]. There were 727 consecutive patients who underwent surgery between January 1997 and August 2011, performed by three surgeons. Of the operations, 32.5 % were performed by surgeon 1, 47.3 % by surgeon 2 and 20.2 % by surgeon 3; 54.2 % of the patients were male. We include age as the only covariate to illustrate how case mix can be incorporated into our model, although a definitive assessment should include more detailed patient characteristics.

The order of operations in each surgeon’s series was used to quantify experience. Our data originated from the national referral centre; hence, we had a record of all operations performed in the UK, ensuring no external experience in this technique was gained; considering different formulations of the experience variable was, thus, not necessary.

The primary outcome considered was in-hospital death, despite it being a rare binary outcome, as PEA is associated with a high risk of adverse events, including post-operative death, and so learning patterns can be observed. A decrease in the proportion of in-hospital deaths signifies an improvement in performance.

#### Limitations of the data

Our dataset had some limitations, which should be considered when undertaking such evaluations.

In the dataset used there were no records of similar operations performed outside the UK by the surgeons involved or of prior training undertaken; however, these cannot be ruled out. Hence, between-surgeon differences in the initial skill level are possible.

A further limitation of this dataset concerns the institutional learning of case selection. Surgeon 1 was the first to undertake the procedure and hence, he was the only attending consultant surgeon. However, surgeon 1 selected the first seven cases to be conducted by the next surgeon joining the team (surgeon 2) and also attended these as supervising surgeon. Surgeon 3, who was the last to take up the procedure, was directly supervised for the whole of his training series, with all his cases preselected by surgeon 2. For all three consultant surgeons, the degree of participation of any additional assisting trainees is not specified and hence, cannot be accounted for.

An issue with non-randomised case series data is that the different case mix treated by each surgeon can potentially mask the true learning effects; this confounding effect may be exacerbated by case selection of the first training cases of surgeons. Ideally, variables that predict patient risk such as age, severity of disease and pre-operative procedure-related measurements should be adjusted for. However, in our dataset there were missing values in most such variables. Our approach allows for case-mix adjustment but the lack of consistently recorded prognostic factors precluded a comprehensive adjustment.

### Model application

**Fig. 2:**
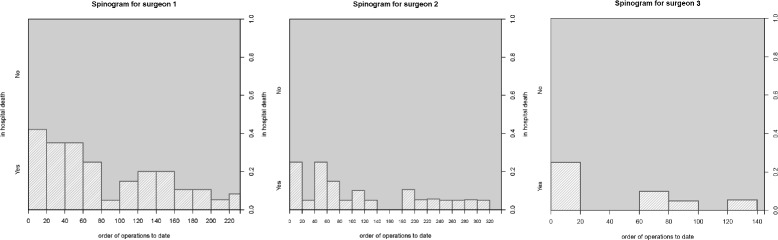
Exploratory analysis of the data structure. Spinograms for **a** surgeon 1, **b** surgeon 2 and **c** surgeon 3

We introduce some exploratory plots to give an idea of the data structure prior to model fitting. The spinograms in Fig. 2 demonstrate that even though surgeon 1’s performance improves, it does not stabilise; there is an indication of a bimodal density. Further examination of this surgeon’s series showed that his practice was interrupted for a period of time, during which some skill was lost. A reduction in in-hospital deaths can be seen for surgeon 2, which seems to stabilise after the 150th operation. For surgeon 3, a steep decrease in in-hospital mortality seems to stabilise by the 25th operation. Notice there is a difference in the learning pattern between surgeons.

The general two-phase model can be expressed algebraically as follows

*Two-phase model:*

1 $$ \phi(p_{ij}) =\left\{ \begin{array}{ll} g(x_{ij};\theta) + {\sum_{k=1}^{w}{\delta_{k}z_{kij}}}, &\quad \text{if}~ x_{ij} < \tau, \\ \alpha + {\sum_{k=1}^{w}{\delta_{k}z_{kij}}}, &\quad \text{if}~ x_{ij} \geq \tau, \end{array}\right.  $$

with *p*_*ij*_ the probability of an in-hospital death for the *j*th patient treated by the *i*th surgeon; *x*_*ij*_ is the operation order and *z*_*kij*_ denote any covariates we wish to include. The parameter *τ* represents the time at which the learning phase ends. The model is fitted using optim() in R; initial values must be supplied. A detailed description of the model’s formulation is given in Additional file 1.

#### Modelling the learning phase

We compared four candidate functional forms on each surgeon’s series to identify the one best suited to the PEA data [7]. Linear and logarithmic functional forms were selected as they are the most commonly used in health care (see Table 1) [7]. The power curve (with non-zero asymptote) was further implemented as it is the most cited in other scientific fields [16] and it suits hierarchical learning structures such as surgery, where routinely applied basic skills are taught first, followed by advanced techniques required for emergency rare cases [17]. Finally, the exponential model (with non-zero asymptote) was fitted as it has been recommended for analysing individual series [17].

**Table 1:** Functional forms under comparison

	*g*(*x*_*ij*_;*θ*)	*θ*	Model constraints
Linear	*β* *x*_*ij*_	(*β*)	*β*≤0
Logarithmic	*β*log(*x*_*ij*_+1)	(*β*)	*β*≤0
Power	*β*(*x*_*ij*_+1)^−*γ*^	(*β*,*γ*)	*β*≥0, *γ*>0, *α*≠0
Exponential	*β*exp(−*γ* *x*_*ij*_)	(*β*,*γ*)	*β*≥0, *γ*>0, *α*≠0

For each surgeon’s curves, we used the Akaike and Bayesian information criteria (AIC and BIC, respectively) as measures of model fit. In this dataset, the linear and logarithmic models seem to fit best, based on the BIC, for all surgeons (see Table 2). However, in these models, the final skill level after large numbers of operations corresponds to an approximately zero event probability, which is unrealistic. Hence, despite their increased complexity, we prefer the three-parameter power and exponential models since they allow for more plausible non-zero asymptotes. The power and exponential models fit equally well based on BIC; thus, to choose between the two we reviewed the parameter estimates for each model, compared to the observed data. Both models converge to $\log \left (\frac {p}{1-p} \right) = \alpha $ with increasing operation order and consequently, the final skill level can be estimated using $p = \frac {e^{\alpha }}{1+e^{\alpha }}.$

**Table 2:** Comparisons between linear, logarithmic, power and exponential models

	Surgeon 1		Surgeon 2		Surgeon 3	
Model	AIC	BIC	AIC	BIC	AIC	BIC
Linear	215.77	226.06	178.61	190.04	63.63	72.52
Logarithmic	216.38	226.66	177.95	189.38	58.83	67.72
Power	218.65	232.37	180.43	195.67	59.63	71.48
Exponential	217.18	230.90	179.02	194.25	58.51	70.36

Note that independent of a surgeon’s skill, a perfect performance level, represented by zero in-hospital deaths, is not plausible. The asymptote estimates are shown in Table 3. Note that these estimates were calculated by averaging the *α* estimates obtained by fitting the models with different initial values, since varying the initial values had a minor influence on estimates.

**Table 3:** Final performance estimates from the simple power and exponential models

	Model	$\hat {\alpha }$	${\exp (\hat {\alpha })}/(1 + \exp (\hat {\alpha }))$
Surgeon 1	Exponential	−2.626	0.067
	Power	−18.713	7.47×10^−9^
Surgeon 2	Exponential	−3.325	0.035
	Power	−8.334	2.40×10^−4^
Surgeon 3	Exponential	−3.984	0.018
	Power	−4.859	0.008

For all surgeons, the power model asymptote estimates are unrealistically small. The estimates from the exponential model for surgeons 2 and 3 fall in the range suggested by experts (0.02–0.05). For surgeon 1, the estimate is slightly higher (0.067) but is still reasonable as the exploratory plots indicate the asymptote was not reached during his observed series.

In summary, for this dataset the power model under-predicted final performance, both when short observed series prevented reliable estimation of asymptotic performance, and also in the remaining cases, indicating that this model was inadequate for describing late-stage performance. Since parameters in these non-linear functions may be correlated, under-prediction of the asymptote may distort other parameter estimates. Thus, even though the BIC difference between the models is minimal, we prefer the exponential model. This is consistent with evidence from the non-health technologies literature, which recommends the exponential model for modelling individual learning curves [17].

## Results

We implemented a negative exponential *β* exp(−*γ*(*x*_*ij*_−*τ*)) for the learning phase of the two-phase model, adjusted for age (centred by the average age treated by each surgeon). To satisfy the continuity constraint at the intersection between the two phases, we use *β* to model the second phase. The constraints *γ*>0 and *τ*≥0 are enforced. The final model fitted is

2 $$ {\begin{aligned} \operatorname{logit}(p_{ij}) =\left\{ \begin{array}{ll} \beta \exp(-\gamma (x_{ij} -\tau)) + \delta\cdot ({age}_{ij}-\overline{age}_{i}), &\quad \text{if } x_{ij}<\tau, \\ \beta + \delta\cdot ({age}_{ij}-\overline{age}_{i}), &\quad \text{if } x_{ij}\geq\tau. \end{array}\right. \end{aligned}}  $$

Selection of initial values was based on both expert opinion and estimates from fitting the three-parameter models. For the duration of learning *τ*, we get intuition from exploratory methods.

**Fig. 3:**
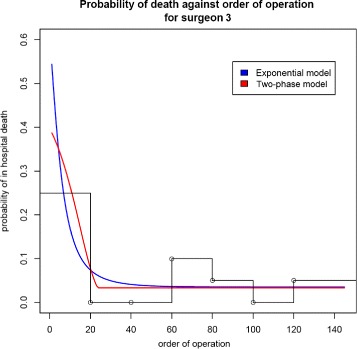
Two-phase and exponential fitted models for surgeon 3

Figure 3 is a plot of the predicted values from fitting the two-phase and exponential models for surgeon 3 for a patient of average age, along with the observed proportion of deaths (in blocks of 20 operations). From Table 4, the learning duration is estimated at 21 operations. The gradients evaluated at the maximum likelihood (ML) estimators are practically zero, confirming that a maximum has been identified. We compare the two-phase to the exponential model. The preferred model is determined on the basis of both model fit and interpretational value. The difference in their BIC values was −1.042, weakly supporting the two-phase model. Since the two-phase model additionally provides an estimate of the learning period duration, it is preferred. The profile likelihood for *τ* is given in Additional file 2.

**Table 4:** Two-phase model for surgeon 3 including age (centred)

Parameter	Estimate	95 % confidence interval
$\hat {\beta }$	−3.998	(−5.350,−2.646)
$\hat {\gamma }$	−0.120	(−0.241,0.001)
$\hat {\tau }$	20.999	(20.133, 21.866)
$\hat {\delta }$	0.094	(0.013, 0.176)
Diagnostics	*AIC*	57.637
	*BIC*	69.489

**Fig. 4:**
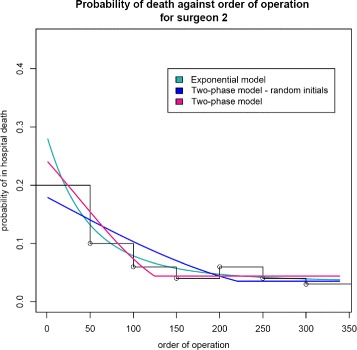
Two-phase and exponential fitted models for surgeon 2

For surgeon 2 there are problems of identifiability with different starting values leading to different estimates. If the initialising *τ*_0_ is chosen in the range 200–220, the model does not move far from these values. This is possibly because we are already on the asymptote so the second phase does describe all data beyond *τ*_0_, which is then adopted as a suitable split point. However, starting from smaller *τ*_0_ (e.g. 50), following a more data-driven *τ*_0_ selection, results in convergence to lower values and better BICs. The estimated split point is *τ*=125, which agrees with the observed data (Fig. 4). Note that whatever our initial choice for *τ*_0_, the estimate to which *τ* converges is never less than 100, indicating that the learning period for surgeon 2 is longer than for surgeon 3.

**Fig. 5:**
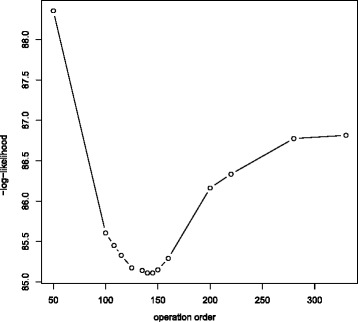
Profile likelihood (*τ*) for surgeon 2

We use the profile likelihood for *τ* to investigate the possibility of multiple turning points. The − log likelihood is plotted to look for a minimum at the ML estimators. From Fig. 5, the global minimum is achieved at approximately operation 140. The profile likelihood tends towards a second turning point, which begins around operation 200, a maximum; unfortunately, the series is too short to observe if indeed a maximum is reached or it remains stable. This change in performance is likely due to a shift in patient case mix treated by surgeon 2 due to surgeon 3 starting to operate around operation 200. Subsequent discussions with the expert surgeons revealed that from there onwards surgeon 2, as the more experienced operator, took over the higher-risk cases and selected the ones to be left as training cases for surgeon 3. Hence, the rapid learning demonstrated by surgeon 3 could be a result of dealing with an easier case mix than surgeon 2 when he started operating; only the first seven cases of surgeon 2, in contrast to more than 50 for surgeon 3, were selected and supervised by a more experienced surgeon. Hence, he had to complete his learning on higher-risk cases, making his progress appear slower. Figure 5 shows it is that performance drop, signifying a change in the mode of learning, which causes the identifiability issues. Evidence that most learning and the principal change in performance occurred by the 125th operation stems from noting the gradient at the ML estimators as well as that between-parameter correlations are worse for higher *τ*_0_.

Consequently, we consider the asymptote was reached at the 125th operation and any changes in performance thereafter are not due to a change in expertise but due to a change in case mix treated. Ideally, one would confirm this by accounting for confounding factors in the analysis; for a prospectively collected case series, we recommend the inclusion of patient and disease characteristics to allow for sufficient case-mix adjustment. The BIC difference between the two-phase logistic and the exponential logistic models is −0.656, weakly supporting the two-phase model. Since the two-phase and exponential models have similar fit statistics but the two-phase model also estimates the learning period duration, one should prefer it.

**Fig. 6:**
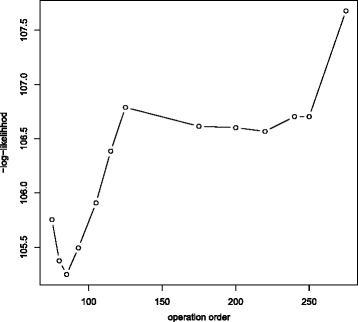
Profile likelihood (*τ*) for surgeon 1

Substantial convergence issues present for surgeon 1 and the model seems likely to converge to two different sets of estimates. From the profile likelihood for *τ* in Fig. 6, two minima can be discerned. A likely explanation is that surgeon 1 stopped performing the operation for four years, possibly resulting in a loss of skill which, even though not so acute as to cause a return to the initial skill level, induced a drop in performance. Upon resuming operations, the surgeon had once more to undergo a learning period. The set of estimates with smaller *τ* includes the final skill level achieved and the time required to reach it for the first performance cycle whereas the set with larger *τ* includes the respective parameters for the second cycle. Parameter estimates are not very robust as instead of one long uninterrupted operation series, we observed two shorter ones, and each on its own was not sufficient to reach the plateau in skill. Better correlations and gradients do occur with *τ* close to the primary learning phase, suggesting most learning was done during the first cycle. Neither model is likely to provide a good fit in this situation and a model with three phases (initial learning, learning after the interruption in practice and post-learning) could be explored.

## Discussion

The novel contribution to the learning curve methodology in this study was to develop and fit a model that not only represents the distribution of the underlying learning data but can also be used to extract estimates of the main learning curve characteristics. Our method allows individual surgeon differences to be explored and can be easily extended to adjust for confounding factors.

For surgeon 1, there were probably two learning periods and a single functional form is unlikely to be adequate in this situation. We suggest fitting a model with phases describing the two learning periods, possibly with some shared parameters, and an additional phase representing the final skill level achieved. The drawback is that robustly fitting such a model requires a large amount of data for each learning period, which is challenging to obtain, especially if dealing with rare dichotomous outcomes. We could not test this multiple-phase model for PEA due to data constraints.

It would be fruitful to investigate if such a loss of skill is also observed in other settings, i.e. with different operation types and surgeons. If this is the case, after completing a predefined training scheme, a surgeon should be randomised as soon as possible in order to avoid losses in skill. In addition, when establishing expertise, simply considering the number of past procedures a surgeon had undertaken would not be sufficient; the time elapsed since that experience was gained should also be considered.

Both the fastest learning rate and the shortest learning period were observed for surgeon 3, the last to take up the intervention. Further discussions with the participating surgeons revealed the existence of institutional learning as those who joined the team at later stages, were directly supervised for at least part of their learning process. This finding is consistent with a group learning effect; as the environment within which the surgeons operate evolves and gains experience of the procedure and surrounding care, improvements in the learning rates of surgeons are anticipated. The same phenomenon was reported in other, non-health technology areas [18]. This shows that the improvement in observed performance is not solely related to greater surgeon skill and experience, but incorporates a range of improvements in multiple components of this complex intervention. In that respect the learning curve attributed to a surgeon is an inaccurate term and it should be interpreted in a much wider sense.

In addition, it is likely that the training cases of newly joined surgeons are selected by the more experienced surgeons to be of low to moderate difficulty. This highlights the importance of comprehensively accounting for patient case mix at the learning curve assessment. It would be undesirable to conclude a surgeon is still on their learning curve simply because they are dealing with more high-risk cases. Likewise, care must be taken not to fall into a false sense of security that a surgeon has completed their learning curve due to only treating very low risk cases.

Although the two-phase model provides maximum information, careful thought should be put into the choice of initial values. Due to its non-linear form, between-parameter correlations are expected and must be accounted for to prevent convergence and discontinuity issues. The choice of initial values should be both expertise-based and data-driven. We recommend, as standard practice, the investigation of the data by exploratory methods and the use of expert opinion to inform the choice of initial values. Pinheiro et al. have also suggested that the choice of initial values for non-linear models should have meaningful graphical interpretations [19]. Both the model fit and its sensitivity to the choice of initial values should be investigated. Model selection should be based on a balance between the criteria indications and the ease of interpretation of the model.

In general, we recommend a dynamic approach to the analysis of learning curves. Initially, simple methods aimed solely at the identification of learning curves should be used. If learning effects are detected, more sophisticated modelling methods, designed for quantifying learning, should be explored; any intuition on the likely shape or parameters of the curve, obtained from the detection methods, should be used to enhance the quantification methods.

### Implications for trial design

Estimation of the learning period duration is frequently hindered by short surgical case series, which do not allow robust fitting of the two-phase model. Cases where the plateau in skill has not been reached during the observed series are quite common [13]. Ideally, long uninterrupted series are needed to explore the evolution of learning over time and as much data as possible should be collected after the asymptote is reached and included in the analysis. Definitive RCTs cannot be greatly extended or recruit large patient numbers for this purpose, due to both financial and ethical reasons [5, 20]. We recommend incorporating the proposed learning curve methodology in the late Phase I and Phase II stages of assessment, which provide suitable settings for learning curve modelling, in preparation for a definitive RCT. At Phase Ib, retrospective case series studies are often undertaken, which even though of low value for technical evaluations, can be used for a primary assessment of learning [5]. They should be conducted on consecutive cases without omissions and use a standardised reporting protocol. Although participating surgeons are limited to a handful of experts at this stage, useful indications of the duration of training required can be derived.

Phase II is pivotal for learning effects modelling. Designs including multiple professionals and hospitals are employed and thus, differential learning patterns amongst surgeons are expected. Since this setting is more representative of the surgeon population that will ultimately deliver the intervention, it is important to assess formally the extent of learning at this stage. We may incorporate learning curve modelling via prospective thoroughly planned studies in combination with smaller feasibility studies usually employed at this phase. Such studies are only viable if prospective research databases recording well-defined outcomes are established [10].

A preliminary Phase I learning curve assessment can readily inform the more valuable Phase II evaluation. The initial values choice could be informed by the learning parameters already observed and a first indication of a functional form appropriate for modelling the innovation under study would be obtained. Finally, the additional practice and training of surgeons participating in both phases would be recorded and thus accounted for at the Phase II learning curve assessment.

Ultimately, we suggest using the data obtained from Phase II to establish the time required by each surgeon to overcome learning. We recommend fitting the two-phase model on the individual surgeons’ series at Phase II to establish when they reach their asymptote and thus, the optimal point for their randomisation to a definitive trial. Since it is expected that different surgeons will reach different final performance levels, an expert level beyond which surgeons are considered sufficiently experienced for trial participation should be predefined; individuals who reach a suboptimal plateau may not be randomised in a definitive RCT until they have undergone additional training and improvement. Finally, surgeons will reach the asymptote at different times due to differential speeds of learning; the definitive RCT should only be initiated when an adequate number of surgeons is sufficiently expert for randomisation.

## Conclusions

The evaluation of complex surgical interventions in RCTs is often complicated due to the existence of operator learning curves. Current approaches accounting for learning curves are for the most part exploratory or descriptive, and only allow the detection but not the quantification of learning. Formal statistical modelling approaches are scarcely used and only model three learning curve features: the initial skill level, the learning rate and the final skill level achieved. In this article, we introduced a fourth feature, the duration of the learning period, and proposed the two-phase model to estimate simultaneously all four learning curve characteristics. We demonstrated how the novel two-phase model could be applied to obtain estimates of the duration of the learning period using an example from cardiac surgery.

This modelling extension is useful as it provides a measure of the potential cost of learning the intervention and enables statisticians to accommodate cases undertaken during the learning phase and assess the intervention after the optimal skill level is reached. We further recommend that learning curve modelling is incorporated both at the late Phase I and Phase II stages of assessment. A preliminary Phase I assessment could directly inform the modelling approach employed for the more extensive Phase II assessment. Finally, the two-phase model could be applied at Phase II to model the individual operators’ learning curves comprehensively and thus, inform the optimal timing of initiation of a definitive RCT.

## Abbreviations

AIC, Akaike information criterion; BIC, Bayesian information criterion; ML, maximum likelihood; PEA, pulmonary endarterectomy; RCT, randomised controlled trial.

## Additional files

Additional file 1 Two-phase model formulation. A detailed description of the formulation and fitting process (via ML estimation) of the two-phase model for both a continuous and a binary outcome. (PDF 174 kb)

Additional file 2 Profile likelihood (*τ*) for surgeon 3. Profile likelihood of *τ* from the two-phase model fitted on surgeon 3’s series. (PDF 12 kb)
