# Supplementary material for: Assessment of learning curves in complex surgical interventions: a consecutive case-series study
Source: Trials. 2016 Jun 1;17:266. doi: 10.1186/s13063-016-1383-4 (PMC4888720; doi:10.1186/s13063-016-1383-4)
Supplement: Additional file 1 — Two-phase model formulation. A detailed description of the formulation and fitting process (via ML estimation) of the two-phase model for both a continuous and a binary outcome. (PDF 174 kb) [file 13063_2016_1383_MOESM1_ESM.pdf]

Two-phase model formulation

In what follows, we give the details of the model formulation for a binary and a continuous outcome respectively.

Generalised non-linear model for a binary outcome

The binary outcome is denoted by y_{ij}, with indices i and j corresponding to surgeon number and operation order respectively. Operation order is determined by ordering each procedure within a surgeon's series by the date conducted. Since our outcome is dichotomous, y_{ij} takes the values 0 for no event and 1 for an event/failure. The generalised and two-phase models are specified as follows:

Generalised non-linear model:

$$\phi(p_{ij}) = \alpha + g(x_{ij};\theta) + \sum_{k=1}^{w} \delta_k z_{kij} \tag{1}$$

Two-phase model:

$$\phi(p_{ij}) = \begin{cases} g(x_{ij};\theta) + \sum_{k=1}^{w} \delta_k z_{kij} & if\ x_{ij} < \tau \\ \alpha + \sum_{k=1}^{w} \delta_k z_{kij} & if\ x_{ij} \geq \tau \end{cases} \tag{2}$$

where the event is assumed to follow a Bernoulli distribution such that $y_{ij} \sim Bernoulli(p_{ij}),\ i = 1,2,3,\ j = 1,...,n_i,\ p_{ij}$ is the probability of event for the jth operation performed by the ith surgeon and n_i the total number of operations performed by the ith surgeon. Experience, denoted by x_{ij}, is the order of the jth operation performed by the ith surgeon i.e. $x_{ij} = j$ from the ith surgeon's series; z_{kij} represents the kth covariate for the jth operation performed by the ith surgeon and w is the total number of covariates. $\phi(.)$ is the link function and since the response variable is binary, the respective canonical link function i.e. the logistic $logit(p_{ij}) = log(\frac{p_{ij}}{1-p_{ij}})$ is preferred. $g(.)$ is a candidate (linear or non-linear) function considered best for describing the learning data. Finally, θ is a scalar or vector of parameters related to the experience covariate and τ is the duration of learning parameter. Models 1 and 2 are fitted via ML estimation. The detailed procedure is as follows.

Recall a Bernoulli likelihood for the jth operation of the ith surgeon is:

$L(y_{ij}) = p_{ij}^{y_{ij}}(1-p_{ij})^{1-y_{ij}}$ and, from model 1 using the $logit$ link function we get $p_{ij} = \frac{\exp(\alpha+g(x_{ij};\theta)+\sum_{k=1}^{w}\delta_k z_{kij})}{1+\exp(\alpha+g(x_{ij};\theta)+\sum_{k=1}^{w}\delta_k z_{kij})}$.

We denote the full vector of parameters as $\theta' = (\theta, \delta_1, ..., \delta_w)$; the respective log-likelihood is $\ell_{ij}(\theta') = y_{ij}log(p_{ij}) + (1-y_{ij})log(1-p_{ij})$.

By independence, the log-likelihood for the full series of the ith surgeon is:

$$\ell_i(\theta') = \sum_{j=1}^{n_i} [y_{ij}log(p_{ij}) + (1-y_{ij})log(1-p_{ij})]$$

$$= \sum_{j=1}^{n_i} [y_{ij}log(\frac{p_{ij}}{1-p_{ij}}) + log(1-p_{ij})]$$

$$= \sum_{j=1}^{n_i} [y_{ij}(\alpha + g(x_{ij};\theta) + \sum_{k=1}^{w} \delta_k z_{kij}) - log(1 + \exp(\alpha + g(x_{ij};\theta) + \sum_{k=1}^{w} \delta_k z_{kij}))]$$

Thus, the log-likelihood to be maximised is:

$$\ell_i(\theta') = \alpha \sum_{j=1}^{n_i} y_{ij} + \sum_{j=1}^{n_i} y_{ij} g(x_{ij};\theta) + \sum_{j=1}^{n_i}\sum_{k=1}^{w} y_{ij}\delta_k z_{kij} - \sum_{j=1}^{n_i} log(1 + \exp(\alpha + g(x_{ij};\theta) + \sum_{k=1}^{w} \delta_k z_{kij}))$$

For the two-phase model this becomes:

$$\ell_i(\theta') = \alpha \sum_{j:x_{ij}\geq\tau} y_{ij} + \sum_{j:x_{ij}<\tau} y_{ij} g(x_{ij};\theta) + \sum_{j=1}^{n_i}\sum_{k=1}^{w} y_{ij}\delta_k z_{kij} - \sum_{j:x_{ij}<\tau} log(1 + \exp(g(x_{ij};\theta) + \sum_{k=1}^{w} \delta_k z_{kij}))$$

$$- \sum_{j:x_{ij}\geq\tau} log(1 + exp(\alpha + \sum_{k=1}^{w} \delta_k z_{kij}))$$

The score vector for the Generalised Non-Linear Model is equivalent to the gradient vector $U = (\frac{\partial\ell_i(\theta')}{\partial\alpha}, \frac{\partial\ell_i(\theta')}{\partial\theta}, \frac{\partial\ell_i(\theta')}{\partial\delta_k})$ for all k and all elements in θ.

$$\frac{\partial\ell_i(\theta')}{\partial\alpha} = \sum_{j=1}^{n_i} y_{ij} - \sum_{j=1}^{n_i} \frac{\exp(\alpha + g(x_{ij};\theta) + \sum_{k=1}^{w} \delta_k z_{kij})}{1 + \exp(\alpha + g(x_{ij};\theta) + \sum_{k=1}^{w} \delta_k z_{kij})}$$

$$\frac{\partial\ell_i(\theta')}{\partial\theta} = \sum_{j=1}^{n_i} y_{ij} g'(x_{ij};\theta) - \sum_{j=1}^{n_i} g'(x_{ij};\theta) \frac{\exp(\alpha + g(x_{ij};\theta) + \sum_{k=1}^{w} \delta_k z_{kij})}{1 + \exp(\alpha + g(x_{ij};\theta) + \sum_{k=1}^{w} \delta_k z_{kij})}$$

$$\frac{\partial\ell_i(\theta')}{\partial\delta_k} = \sum_{j=1}^{n_i} y_{ij} z_{kij} - \sum_{j=1}^{n_i} z_{kij} \frac{\exp(\alpha + g(x_{ij};\theta) + \sum_{k=1}^{w} \delta_k z_{kij})}{1 + \exp(\alpha + g(x_{ij};\theta) + \sum_{k=1}^{w} \delta_k z_{kij})}$$

Setting the above partial derivatives to zero and solving them simultaneously gives the ML estimators of the parameters.

This estimation process was implemented in the statistical programming language R 3.0.1 using the optimisation command *optim()* , selecting a quasi-Newton method also known as a variable metric algorithm.

Generalised non-linear model for a continuous outcome

A continuous outcome variable is denoted by y_{ij}, with indices i and j corresponding to surgeon number and operation order respectively. The generalised and two-phase models are specified as follows.

Generalised non-linear model:

$$y_{ij} = \alpha + g(x_{ij};\theta) + \sum_{k=1}^{w} \delta_k z_{kij} + e_{ij} \tag{3}$$

Two-phase model:

$$y_{ij} = \begin{cases} g(x_{ij};\theta) + \sum_{k=1}^{w} \delta_k z_{kij} + e_{ij} & if\ x_{ij} < \tau \\ \alpha + \sum_{k=1}^{w} \delta_k z_{kij} + e_{ij} & if\ x_{ij} \geq \tau \end{cases} \tag{4}$$

with the errors following a Normal distribution $e_{ij} \sim Normal(0, \sigma^2)$, $i = 1, 2, 3,\ \ j = 1, ..., n_i$. Hence, the continuous variable is assumed to follow a Normal distribution such that $y_{ij} \sim Normal(\mu_{ij}, \sigma^2)$, $i = 1, 2, 3,\ \ j = 1, ..., n_i$ and $\mu_{ij} = \alpha + g(x_{ij};\theta) + \sum_{k=1}^{w} \delta_k z_{kij}$. In the two-phase model, at the first phase $\mu_{ij} = g(x_{ij};\theta) + \sum_{k=1}^{w} \delta_k z_{kij}$ and at the second phase, $\mu_{ij} = \alpha + \sum_{k=1}^{w} \delta_k z_{kij}$. Experience is represented by x_{ij}, the order of the jth operation performed by the ith surgeon; z_{kij} represents the kth covariate for the jth operation performed by the ith surgeon and w is the total number of covariates. $g(.)$ is a candidate (linear, or non-linear) function considered for describing the learning data best. Finally, θ is a scalar or vector of parameters related to the experience covariate and τ is the duration of learning parameter. Models 3 and 4 are fitted via ML estimation. The detailed procedure is as follows.

Recall a Normal likelihood for the jth operation in the ith surgeon's series is:

$$L(y_{ij}) = \frac{1}{\sqrt{2\pi\sigma^2}} \exp\left(\frac{-1}{2\sigma^2}(y_{ij} - \mu_{ij})^2\right)$$

$$L(y_{ij}) = \exp\left(\frac{y_{ij}\mu_{ij} - \mu_{ij}^2/2}{\sigma^2} - \frac{1}{2}\log 2\pi - \log\sigma - \frac{y_{ij}^2}{2\sigma^2}\right)$$

We denote the full vector of parameters as $\theta' = (\theta, \delta_1, ..., \delta_w, \sigma^2)$; the respective log-likelihood is $\ell_{ij}(\theta') = \frac{y_{ij}\mu_{ij} - \mu_{ij}^2/2}{\sigma^2} - \frac{1}{2}\log 2\pi - \log\sigma - \frac{y_{ij}^2}{2\sigma^2}$.

By independence, the joint log-likelihood for the series of the ith surgeon is:

$$\ell_i(\theta') = \sum_{j=1}^{n_i}\left[\frac{y_{ij}\mu_{ij} - \mu_{ij}^2/2}{\sigma^2} - \frac{y_{ij}^2}{2\sigma^2} - \log\sigma - \frac{1}{2}\log 2\pi\right]$$

$$= \sum_{j=1}^{n_i}\left[\frac{y_{ij}(\alpha + g(x_{ij};\theta) + \sum_{k=1}^{w}\delta_k z_{kij})}{\sigma^2} - \frac{(\alpha + g(x_{ij};\theta) + \sum_{k=1}^{w}\delta_k z_{kij})^2}{2\sigma^2} - \frac{y_{ij}^2}{2\sigma^2} - \log\sigma - \frac{1}{2}\log 2\pi\right]$$

$$= \alpha\sum_{j=1}^{n_i}\frac{y_{ij}}{\sigma^2} + \sum_{j=1}^{n_i}\frac{y_{ij}g(x_{ij};\theta)}{\sigma^2} + \sum_{j=1}^{n_i}\frac{y_{ij}\sum_{k=1}^{w}\delta_k z_{kij}}{\sigma^2} - \frac{n_i\alpha^2}{2\sigma^2} - \frac{\alpha}{\sigma^2}\sum_{j=1}^{n_i}\left(g(x_{ij};\theta) + \sum_{k=1}^{w}\delta_k z_{kij}\right)$$

$$-\sum_{j=1}^{n_i}\frac{(g(x_{ij};\theta) + \sum_{k=1}^{w}\delta_k z_{kij})^2}{2\sigma^2} - \sum_{j=1}^{n_i}\frac{y_{ij}^2}{2\sigma^2} - n_i\log\sigma - \frac{n_i}{2}\log 2\pi$$

For the two-phase model, the likelihood becomes:

$$\ell_i(\theta') \propto \sum_{j:x_{ij}<\tau} \left[\frac{y_{ij}(g(x_{ij};\theta) + \sum_{k=1}^{w} \delta_k z_{kij})}{\sigma^2} - \frac{(g(x_{ij};\theta) + \sum_{k=1}^{w} \delta_k z_{kij})^2}{2\sigma^2} - \log\sigma - \frac{y_{ij}^2}{2\sigma^2} \right]$$

$$+ \sum_{j:x_{ij}\geq\tau} \left[\frac{y_{ij}(\alpha + \sum_{k=1}^{w} \delta_k z_{kij})}{\sigma^2} - \frac{(\alpha + \sum_{k=1}^{w} \delta_k z_{kij})^2}{2\sigma^2} - \log\sigma - \frac{y_{ij}^2}{2\sigma^2} \right]$$

This can be split in three parts:

$$\ell_i(\theta') \propto \sum_{j:x_{ij}<\tau} \left[\frac{y_{ij} g(x_{ij};\theta)}{\sigma^2} - \frac{(g(x_{ij};\theta))^2 + 2g(x_{ij};\theta)\sum_{k=1}^{w} \delta_k z_{kij}}{2\sigma^2} \right]$$

$$+ \sum_{j:x_{ij}\geq\tau} \left[\frac{y_{ij}\alpha}{\sigma^2} - \frac{\alpha^2 + 2\alpha\sum_{k=1}^{w} \delta_k z_{kij}}{2\sigma^2} \right]$$

$$+ \sum_{j=1}^{n_i} \left[\frac{y_{ij}\sum_{k=1}^{w} \delta_k z_{kij}}{\sigma^2} - \frac{(\sum_{k=1}^{w} \delta_k z_{kij})^2}{2\sigma^2} - \frac{y_{ij}^2}{2\sigma^2} \right] - n_i \log\sigma$$

The score vector for the Generalised Non-Linear Model (GNLM) is equivalent to the gradient vector $U = (\frac{\partial \ell_i(\theta')}{\partial \alpha}, \frac{\partial \ell_i(\theta')}{\partial \theta}, \frac{\partial \ell_i(\theta')}{\partial \delta_k}, \frac{\partial \ell_i(\theta')}{\partial \sigma^2})$ for all k and all elements in θ.

$$\frac{\partial \ell_i(\theta')}{\partial \alpha} = \sum_{j=1}^{n_i} \frac{y_{ij}}{\sigma^2} - \frac{n_i\alpha}{\sigma^2} - \frac{1}{\sigma^2}\sum_{j=1}^{n_i}\left(g(x_{ij};\theta) + \sum_{k=1}^{w} \delta_k z_{kij} \right)$$

$$= \frac{1}{\sigma^2}\sum_{j=1}^{n_i}\Big(y_{ij} - g(x_{ij};\theta) - \sum_{k=1}^{w} \delta_k z_{kij} \Big) - \frac{n_i\alpha}{\sigma^2}$$

$$\frac{\partial \ell_i(\theta')}{\partial \theta} = \sum_{j=1}^{n_i} \frac{y_{ij} g'(x_{ij};\theta)}{\sigma^2} - \frac{\alpha}{\sigma^2}\sum_{j=1}^{n_i} g'(x_{ij};\theta) - \frac{1}{\sigma^2}\sum_{j=1}^{n_i} g'(x_{ij};\theta)\Big(g(x_{ij};\theta) + \sum_{k=1}^{w} \delta_k z_{kij} \Big)$$

$$= \frac{1}{\sigma^2}\sum_{j=1}^{n_i} g'(x_{ij};\theta)\Big(y_{ij} - \alpha - g(x_{ij};\theta) - \sum_{k=1}^{w} \delta_k z_{kij} \Big)$$

$$\frac{\partial \ell_i(\theta')}{\partial \delta_k} = \sum_{j=1}^{n_i} \frac{y_{ij} z_{kij}}{\sigma^2} - \frac{\alpha}{\sigma^2}\sum_{j=1}^{n_i} z_{kij} - \frac{1}{\sigma^2}\sum_{j=1}^{n_i} z_{kij}\Big(g(x_{ij};\theta) + \sum_{k=1}^{w} \delta_k z_{kij} \Big)$$

$$= \frac{1}{\sigma^2}\sum_{j=1}^{n_i} z_{kij}\Big(y_{ij} - \alpha - g(x_{ij};\theta) - \sum_{k=1}^{w} \delta_k z_{kij} \Big)$$

$$\frac{\partial \ell_i(\theta')}{\partial \sigma^2} = -\frac{1}{\sigma^4}\left[\sum_{j=1}^{n_i} y_{ij}\Big(\alpha+g(x_{ij};\theta)+\sum_{k=1}^{w}\delta_k z_{kij}\Big)-\frac{n_i\alpha^2}{2}-\alpha\sum_{j=1}^{n_i}\Big(g(x_{ij};\theta)+\sum_{k=1}^{w}\delta_k z_{kij}\Big)\right.$$

$$\left.-\frac{1}{2}\sum_{j=1}^{n_i}\Big(\big(g(x_{ij};\theta)+\sum_{k=1}^{w}\delta_k z_{kij}\big)^2-y_{ij}^2\Big)\right]-\frac{n_i}{2\sigma^2}$$

Setting the above partial derivatives to zero and solving them simultaneously gives the ML estimators of the parameters.

This estimation process may be implemented as for the binary outcome case using *optim()* in R.
