# Supplementary figures and images for: Assessment of learning curves in complex surgical interventions: a consecutive case-series study
Source: Trials. 2016 Jun 1;17:266. doi: 10.1186/s13063-016-1383-4 (PMC4888720; doi:10.1186/s13063-016-1383-4)

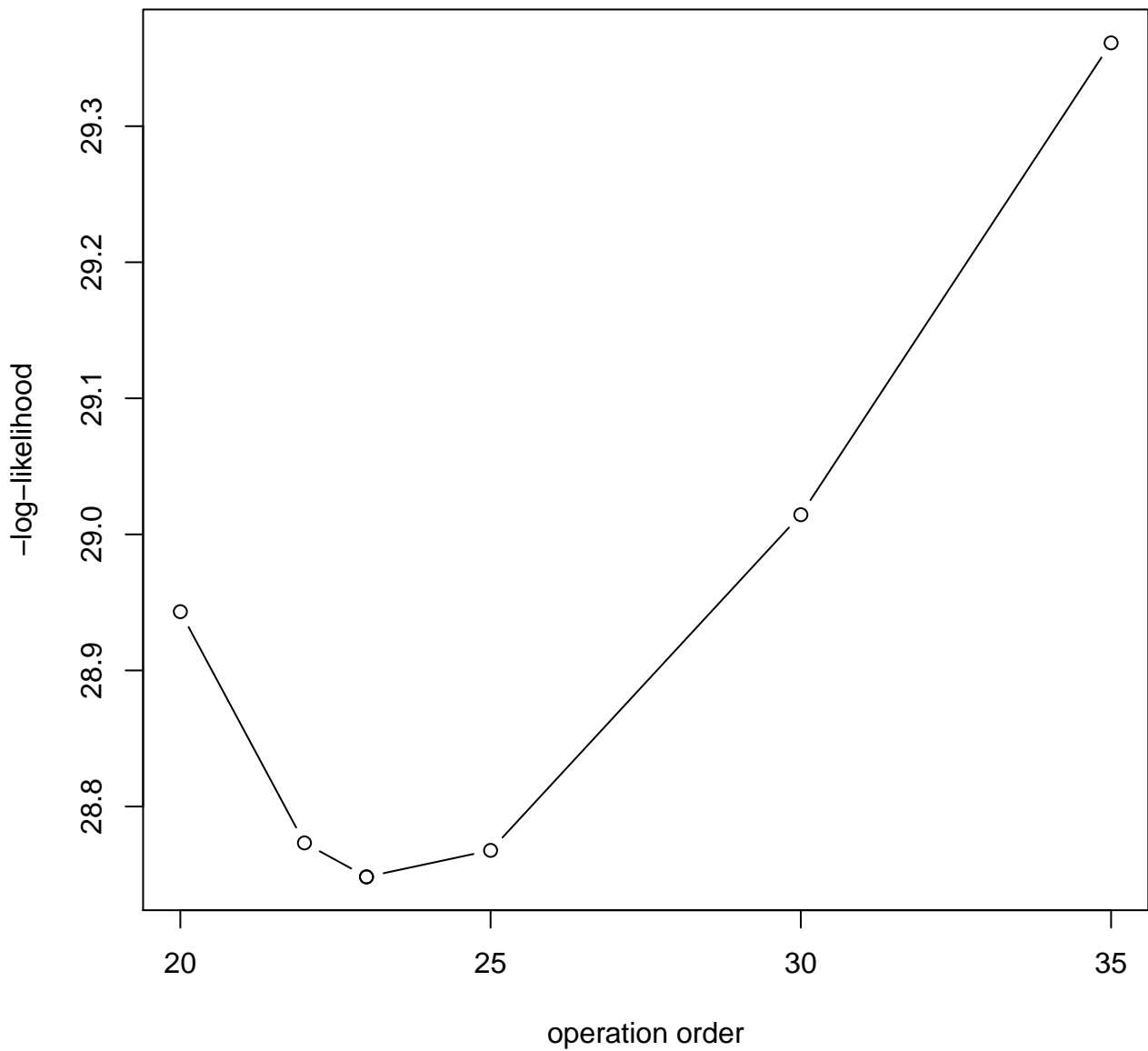

Supplement: Additional file 2 — Profile likelihood (τ) for surgeon 3. Profile likelihood of τ from the two-phase model fitted on surgeon 3’s series. (PDF 12 kb) [file 13063_2016_1383_MOESM2_ESM.pdf]
